# Kinetics and thermodynamics investigations of efficient and eco-friendly removal of alizarin red S from water via acid-activated *Dalbergia sissoo* leaf powder and its magnetic iron oxide nanocomposite

**DOI:** 10.3389/fchem.2024.1457265

**Published:** 2024-09-25

**Authors:** Saleem Nawaz, Syed Muhammad Salman, Asad Ali, Basit Ali, Syed Nusrat Shah, Latif Ur Rahman

**Affiliations:** ^1^ Department of Chemistry, Islamia College Peshawar, Peshawar, Pakistan; ^2^ Energy Engineering, Division of Energy Science, Lulea University of Technology, Lulea, Sweden; ^3^ Institute of Chemical Sciences, University of Peshawar, Peshawar, Pakistan

**Keywords:** alizarin red S, adsorptive removal, *Dalbergia sissoo*, *Dalbergia sissoo*-magnetic iron oxide nanocomposite, adsorption isotherms, thermodynamics and kinetics

## Abstract

The present work aimed to highlight an efficient, readily accessible, and cost-effective adsorbent derived from *Dalbergia sissoo* (DS) leaf powder for removing the environmentally hazardous dye “alizarin red S” (ARS) from hydrous medium. A variant of the adsorbent is activated via sulfuric acid and composited with magnetic iron oxide nanoparticles (DSMNC). Both adsorbents are thoroughly characterized using techniques such as Fourier transform infrared spectroscopy, point of zero charge, energy-dispersive X-ray spectroscopy, and scanning electron microscopy, which show that they have a porous structure rich in active sites. Different adsorption conditions are optimized with the maximum removal efficiency of 76.63% for DS and 97.89% for DSMNC. The study was highlighted via the application of various adsorption isotherms, including Freundlich, Langmuir, Temkin, and Dubinin–Radushkevich, to adsorption data. Pseudo-first-order, pseudo-second-order, and intra-particle diffusion models were utilized to investigate the kinetics and mechanism of adsorption. The Freundlich model and pseudo-second-order kinetics exhibited the best fit, suggesting a combination of physical interactions, as confirmed by the D–R and Temkin models. The dominant adsorbate–adsorbent interactive interactions responsible for ARS removal were hydrogen bonding, dispersion forces, and noncovalent aromatic ring adsorbent pi-interactions. Thermodynamic parameters extracted from adsorption data indicated that the removal of the mutagenic dye “ARS” was exothermic and spontaneous on both DS and DSMNC, with DSMNC exhibiting higher removal efficiency.

## 1 Introduction

The direct release of harmful waste from various origins into aquatic environments that lack adequate processing is the primary challenge to water quality. A significant source of water pollution stems from organic pollutant effluents via numerous industries, including leather manufacturing, paint production, textiles, and paper. Even in trace amounts, these substances can harm aquatic environments and pose risks to all living organisms (Lebkiri et al., 2023; Bensalah et al., 2023). For instance, the dye industry is ranked as the 10th most contaminating sector in terms of river water quality, contributing 17%–20% of industrial water degradation (Kadiri et al., 2021; Gul et al., 2021). Annually, between 5,000 and 10,000 tons of dyes are dumped into rivers owing to their widespread usage. Alizarin red S (ARS) is one of the most commonly used dyes. These dyes are classified as anionic dyes because their molecules dissociate in water with negative charges, posing a risk to the health of the marine habitat and the surrounding community when directly expelled into surface waters. They obstruct the sunlight that is vital for the process of photosynthesis in aquatic plants (Pirkarami and Olya, 2017; Yagub et al., 2014; Wong et al., 2020). Consequently, effective management of textile effluents is crucial to safeguard the ecosystem and the surrounding environment.

Alizarin red S (ARS), also known as 1,2-dihydroxy-9,10-anthraquinone sulfonic acid sodium salt, is a dye that has been used extensively since ancient times and has significant adverse impacts on the ecosystem (Hu et al., 2019). It is highly desired by industries such as textiles, food, and dyes due to its water solubility of 20 g/L (Fayazi et al., 2015). Acting as a color indicator, it has a pKa value ranging from 4.6 to 6.5 and is commonly found in the effluent of these sectors (Chin et al., 2015). Data from tests on rabbits indicate that ARS can sensitize the skin, cause minor irritation to the eyes, and is known to be hazardous and carcinogenic (Sowjanya et al., 2022). It has been shown to induce dermatitis in humans and has the potential to act as an allergen. Its LD_50_ value, when administered intravascularly, is 70 mg/kg (Gautam et al., 2020). Due to its potential to cause oxidative damage to organisms, it has been claimed to be mutagenic and carcinogenic (Delpiano et al., 2021).

Membrane percolation, photolytic decay, high-performance liquid chromatography, membrane electrophoresis, flocculation-coagulation, chemical and electrochemical oxidation, ion exchange processes, filtration followed by coagulation, ozonation followed by coagulation, and adsorption are common techniques for analyzing and treating pollutants (Jebli et al., 2023; Gautam et al., 2017; Kamarehie et al., 2019). However, the extensive use of costly chemicals, poor effectiveness, formation of secondary noxious waste, and high operating and upkeep charges limit their applicability (Detpisuttitham et al., 2020). Compared to other methods, adsorption is highly effective at removing colors from industrial effluents, resulting in clean, high-quality water by eliminating most contaminants found in wastewater.

When treating runoff from the dye industry using an adsorption technique, pure, high-quality water free of coloring material and other contaminants is produced. Despite extremely small concentrations, the presence of dyes can diminish the visual appeal of water. Due to the durability and complex aromatic ring structure of dyes, persistent dyes like ARS are challenging to treat in aqueous mediums using conventional methods. Adsorption, however, offers a simple, cost-effective method that has gained popularity due to its minimal waste disposal advantage (Zhou et al., 2019; Aragaw and Bogale, 2021; Nistor et al., 2021).

According to the literature that has evaluated diverse biosorbents, biosorption is among the most cost-effective and straightforward practices for removing color from industrial effluent (Adegoke and Bello, 2015; Kumar et al., 2021). This method utilizes readily available, reasonably priced, and efficiently treated or untreated materials as biosorbents. Biosorbents can be treated with different acids to enhance their capacity to absorb various colors on their fine, porous surfaces.

The quantity of dye that can be adsorbed depends on several factors, including pH, contact time, temperature, type and dosage of adsorbents, and original dye concentration. The optimal sorbent parameters for specific dye adsorption can vary significantly (Sultana et al., 2023), and optimizing each parameter is beneficial for both large-scale commercial applications and understanding the adsorption mechanism. Colors from industrial runoff can be effectively biosorbed through the utilization of plant biomasses, rice husks, algae, walnut shells, wood, coconut coir dust, and numerous other waste materials that have undergone chemical modification (Kainth et al., 2024).

Adsorptive removal of ARS using *Dilbergia sissoo* leaf powder and its magnetic nanocomposite has not been reported. According to the experimental results, 78.7% of alizarin red S was removed from water-based solutions under optimum environmental factors using polypyrrole-coated magnetic nanoparticles (Gholivand et al., 2015).

In the present study, powdered *Dilbergia sissoo* (DS) leaves underwent treatment with acid and were subsequently composited with magnetic iron oxide nanoparticles to create an adsorbent for the elimination of ARS from an aqueous medium.

## 2 Material and methods

### 2.1 Adsorbent preparation

Leaves of DS were cleansed repeatedly using distilled water to eliminate any grime present. Afterward, the DS sample was oven-dried at 80°C for 24 h and subsequently screened to acquire DS particles with size 125 μm. DS powder was soaked in 2M H_2_SO_4_ (Merck 98%) for 24 h for activation, cleansed with distilled water twice to remove the acid, and dehydrated in an oven at 80°C.

Magnetic iron oxide-nanocomposite was prepared by the co-precipitation method (Oroujizad et al., 2023). In this method, specified quantities (1:2 mole ratio) of FeSO_4_.7H_2_O (Sigma Aldrich ≥99%) and FeCl_3_.6H_2_O (Sigma Aldrich ≥99%) were dispersed in 100 mL of deionized water and continuously stirred at 70–80°C with the continuous dropwise addition of 10% ammonia (Sigma Aldrich 25%) until pH 10 is achieved. Then, the activated DS leaf powder (1g) was slowly added until a black precipitate was formed, indicating the formation of the nanocomposite. The hot mixture containing the precipitate was brought to ambient temperature, filtered, and cleaned with distilled water. The residue was dehydrated at 60°C in an oven and converted to a fine powder form.

### 2.2 Solution preparation

A 1,000 mg/L stock solution of ARS (Sigma Aldrich 97%) was prepared that was subsequently diluted to create desired dye working solutions. HCl (Sigma Aldrich 37%) and NaOH (Merck ≥ 97.0%) solutions having 0.1M concentrations were used to regulate the solution pH.

### 2.3 Characterization of DS and DSMNC

To comprehensively comprehend the adsorption mechanism, the prepared adsorbents underwent characterization both pre- and post-adsorption utilizing Fourier transform infrared spectroscopy (FTIR) (Agilent Technologies, United States), scanning electron microscopy (SEM), and energy-dispersive X-ray spectroscopy (EDS) (JEOL Japan). The adsorption efficiency was determined using a UV-visible spectrophotometer from Shimadzu (model 1900) in Japan.

### 2.4 Batch adsorption analysis

A batch sorption analysis was carried out using a 100 mL conical flask containing 50 mL of ARS solution (40–120 mg.L^−1^) placed on a shaker operating at 200 rpm (15–150 min). After each adsorption experiment, the DS and DSMNC composite (0.05–0.6 g) were separated from the dye effluent through filtration, and the absorbance of each solution was measured at 423 nm (experimentally determined at pH_PZC_) to determine its concentration after adsorption. The percentage of adsorptive removal (E%) and the maximum uptake capacity (q_e_ in mg/g) of ARS were determined using Equations 1,2. These equations utilized an ARS reference curve established with various working solutions of the dye.

$$q_{e}=\frac{\left(C_{0}-C_{a}\right)V}{m},$$ (1)

$$\%Removal=\frac{\left(C_{0}-C_{a}\right)}{C_{0}}\;X\;100,$$ (2)

where C_o_ (mg/L) is the concentration of ARS before adsorption, C_a_ (mg/L) is the ARS concentration after adsorption, V(L) is the volume of solution in liters, and m (g) is the mass of the adsorbent.

Kinetics, isothermal, and thermodynamic models were applied to the adsorption data.

## 3 Result and discussion

### 3.1 Characterizations of adsorbents

**FIGURE 1:**
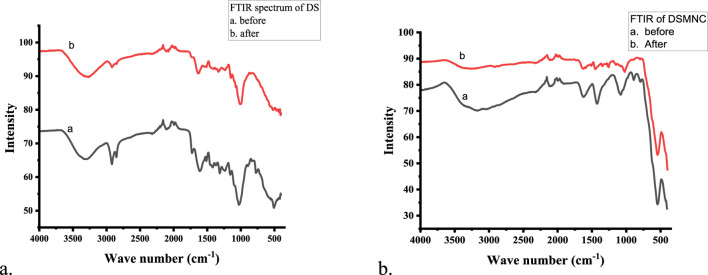
FTIR spectra of **(A)** DS and **(B)** DSMNC before and after adsorption.

The functional groups located on the surface of the adsorbents (DS and DSMNC) were assessed through Fourier transform infrared spectroscopy (FTIR) and are depicted in Figures 1A, B. FTIR spectra revealed the presence of numerous functional groups.

The appearance of a strong peak at 544 cm^−1^, denoting stretching of the Fe-O bond, is confirmation of the formation of magnetic iron oxide nanocomposite (Salari, 2022; Mahmoodi et al., 2019). The occurrence of –OH and –NH functional groups was verified by the bands observed between 3,000 cm^−1^ and 4,000 cm^−1^ (Ali et al., 2019). The peak at 2,850–2,922 cm^−1^ accounts for C-H stretching (Pal et al., 2007). The appearance of the peak at 2,100–2,260 cm^−1^ corresponds to C≡C stretching. The pronounced peaks observed within the wave number range of 1,600–1,650 cm^−1^ indicate the presence of conjugated stretching associated with the carboxylic and carbonyl groups’ C=O bonds (Malik et al., 2020). The peaks between 1,400 cm^−1^ and 1,450 cm^−1^ correspond to the aromatic ring present (Oyekanmi et al., 2021). The peaks between 1,200 cm^−1^ and 1,320 cm^−1^ correspond to a carboxylic acid group. The peaks between 1,020 cm^−1^ and 1,200 cm^−1^ indicate C-OH stretching (Dehkhoda et al., 2014). The peak appearing between 675 cm^−1^ and 895 cm^−1^ corresponds to C=C. Shifting of some of the peaks identified in the FTIR spectra of the dye-loaded samples suggests interactions between the dye molecules and the sorbent material. These groups actively participate in the interactions and hydrogen bonds and enhance the degree of adsorption (Jawad et al., 2020; Oyekanmi et al., 2021).

**FIGURE 2:**
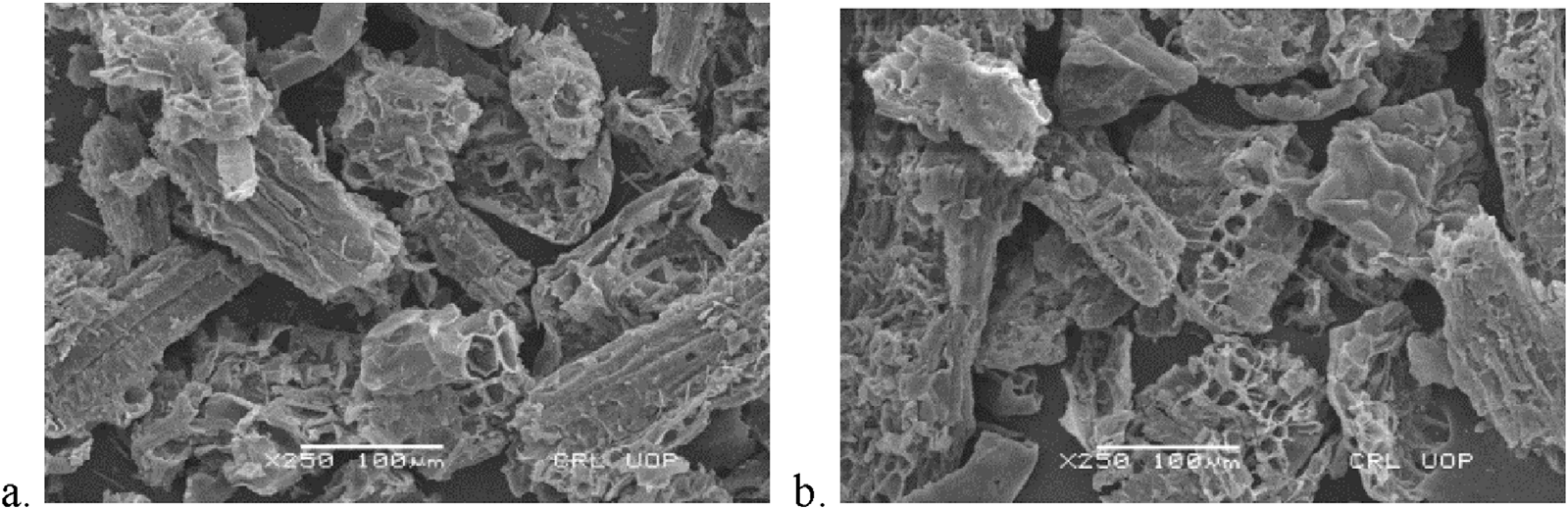
SEM analysis of DS **(A)** before and **(B)** after adsorption.

**FIGURE 3:**
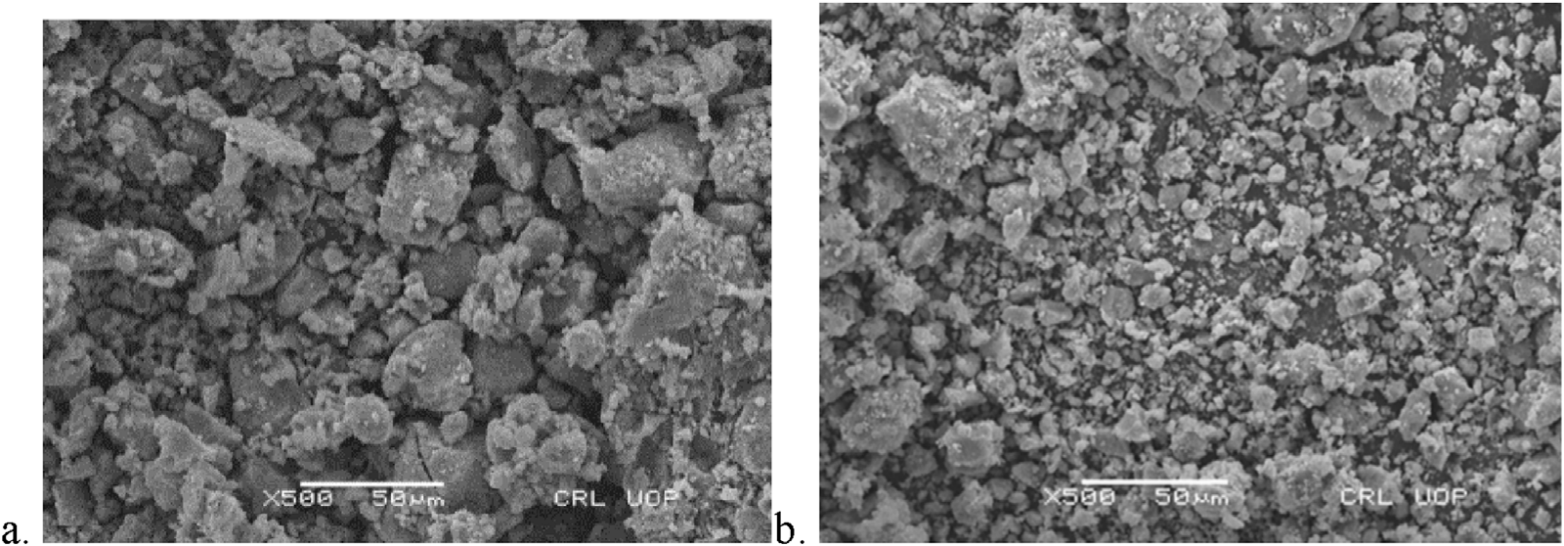
SEM analysis of DSMNC **(A)** before and **(B)** after adsorption.

The surface morphology of the adsorbent is characterized by the SEM images of DS and DSMNC before and after adsorption depicted in Figures 2, 3. It is clear from the figure that the particle size decreases when the magnetic nanocomposite is formed with the biomass. An increase in surface area and an increased amount of adsorbed dye is expected on DSMNC compared to DS.

**FIGURE 4:**
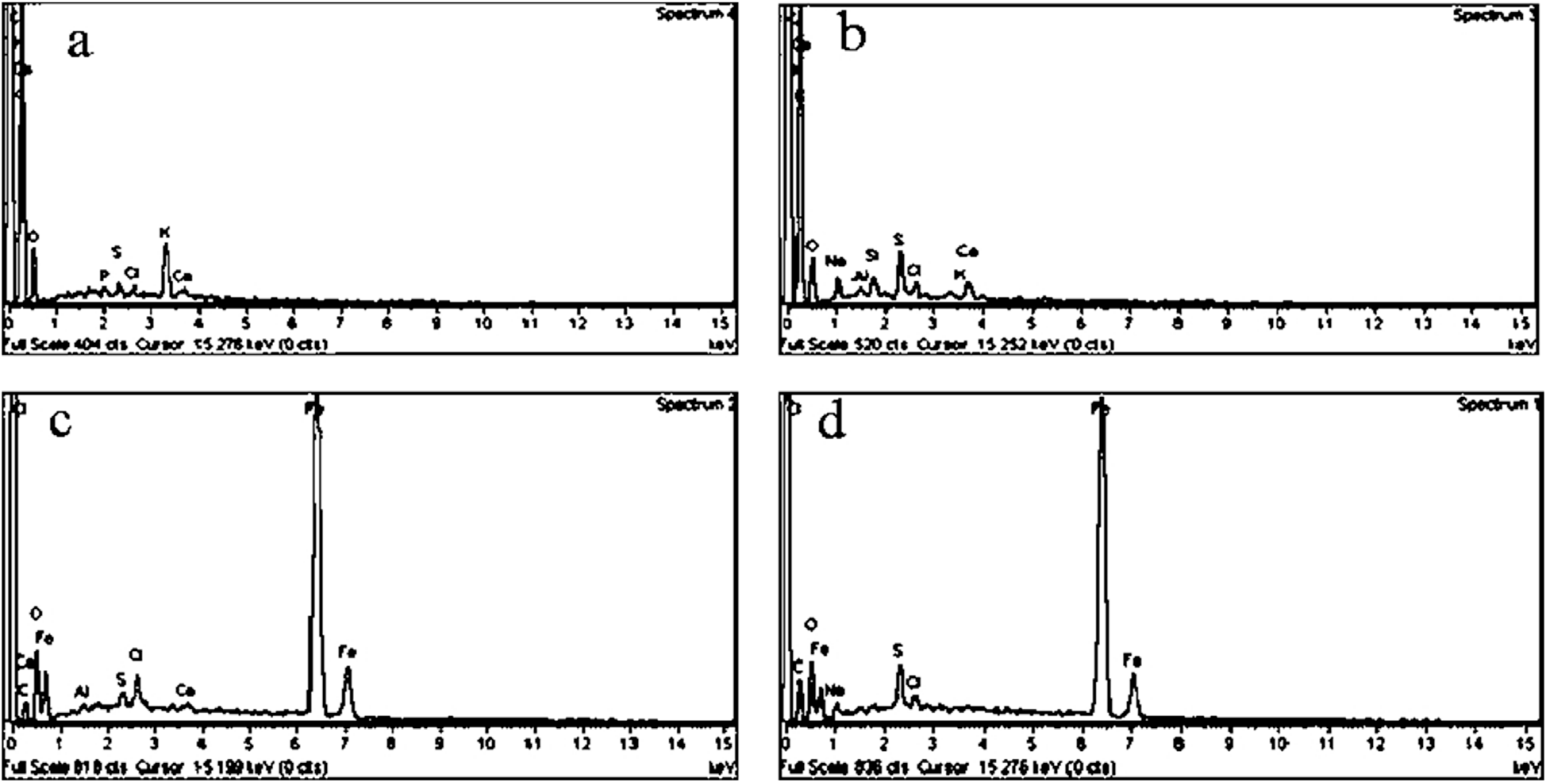
EDS spectra of DS **(A)** before and **(B)** after adsorption and of DSMNC **(C)** before and **(D)** after adsorption.

The EDS spectra of DS and DSMNC are visualized in Figures 4A–D, respectively. The appearance of strong Fe peaks in the EDS spectra of DSMNC confirms the formation of the magnetic nanocomposite. Additionally, a hyperchromic shift is observed in the peaks of C, S, and Na in the post-adsorption spectra of both DS and DSMNC. This shift indicates an increase in the elemental compositions of these elements present in ARS, supporting its adsorption onto both DS and DSMNC.

In the context of studying the sorption of colorant from aqueous media, the pH level at which adsorbent exhibits zero cumulative surface charge, known as the point of zero charge (pH_PZC_), emerges as a critical parameter. The pH level of the dye solution significantly contributes to determining the amount of adsorbent that can be effectively adsorbed (Chowdhury et al., 2011; Murthy et al., 2020). This is because variations in pH facilitate the electrification of both the adsorbate particles and the functional moieties present upon the adsorbent interface; consequently, alterations in the pH of the medium alter the surface charges on the adsorbent, thereby influencing the rate of adsorption (Sen et al., 2011).

**FIGURE 5:**
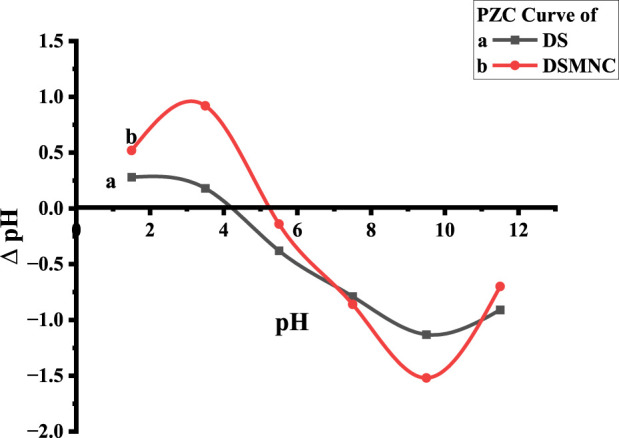
pH_pzc_ of DS and DSMNC.

The pH_PZC_ values obtained from the plot are 5.2 for DS and 4.2 for DSMNC shown by Figure 5. pH values lower than the pH_PZC_ are ideal for the adsorption of ARS with anionic groups. This is because protonation causes the adsorbent surface to become cationic, facilitating the adsorption process.

### 3.2 Optimization of adsorption factors

**FIGURE 6:**
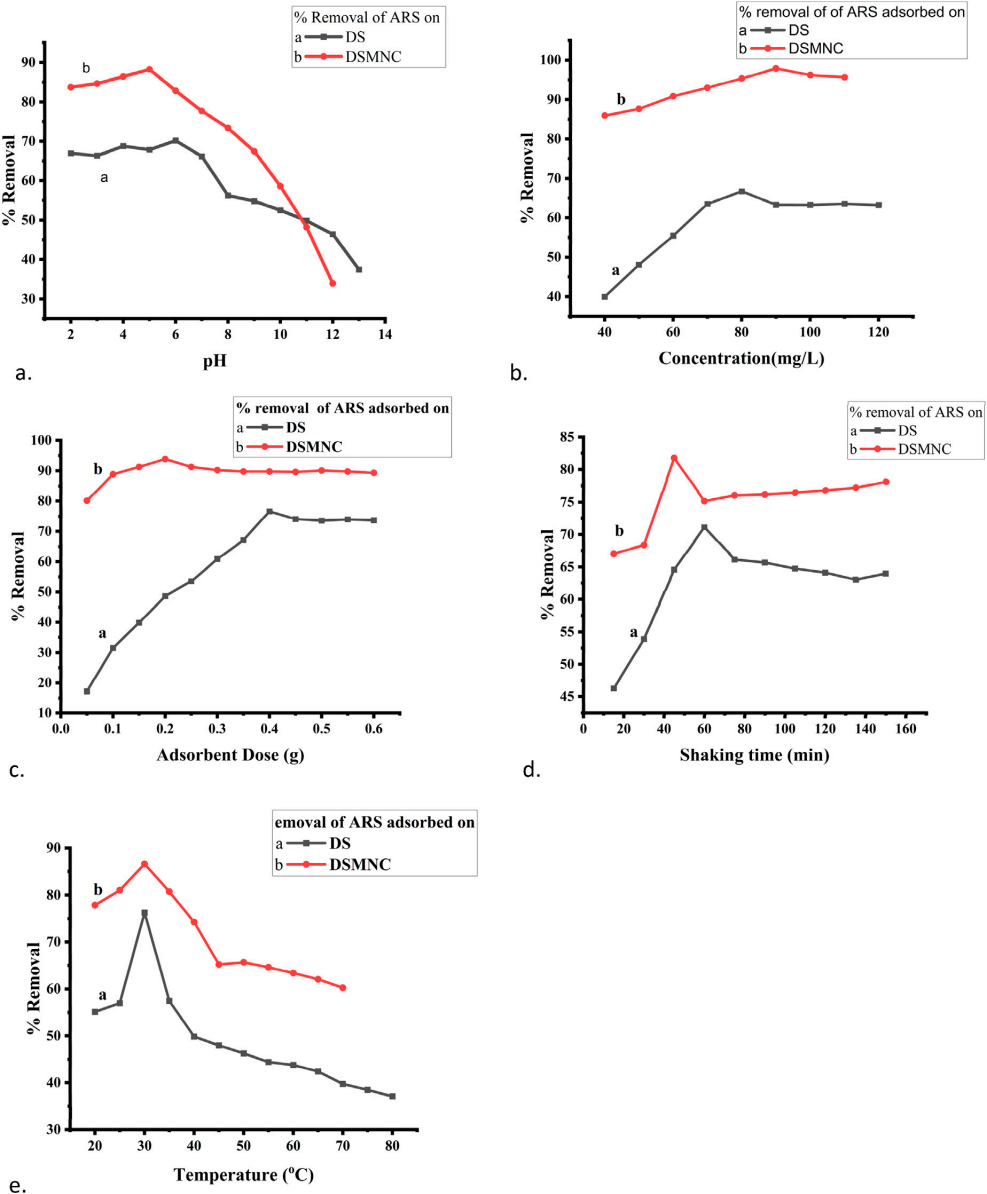
Optimization of adsorption parameters: **(A)** pH, **(B)** concentration, **(C)** adsorbent dose, **(D)** shaking time, and **(E)** temperature for adsorption of ARS on DS and DSMNC.

Figure 6 represents fine-tuning of adsorption conditions for the removal of ARS using DS and DSMNC. A batch adsorption experiment was performed to examine the impact of the adsorbent dose on the removal of ARS onto the surface of DS and DSMNC. The adsorbents (0.05–0.6 g) were added to 50 mL of 100 mg/L of ARS, taken in 250 mL flasks, and shaken for 60 min at 200 rpm. The remaining dye content in the solutions was subsequently determined via spectrophotometry. Similar experimental approaches were employed to scrutinize the influence of the preliminary dye concentration (40 mg/L–120 mg/L), shaking time (15–150 min), pH (1.5–13.5) ionic strength, and temperature (20°C–80°C) on the adsorption process.

The effect of pH is shown in Figure 6A by plotting the percentage removal vs. pH. As shown by the figure, the percentage removal of ARS increases with the increase of pH, reaches the optimum value near pH_PZC_, and decreases afterward at a constant amount of adsorbent dose, concentration, temperature, and shaking time. The removal efficiency is higher when DSMNC (88.25%) is used than when DS (70.2%) is used as an adsorbent. The values of q_e_ remain almost constant till pH_PZC_ and are followed by a decrease above this pH. The surplus positive charges will additionally impact the surface charge of the adsorbent. Consequently, the intricately structured aromatic compounds of ARS will progressively adhere to the highly porous surface of the adsorbent, thereby stabilizing the dispersion status (Zare et al., 2015).

The influence of the initial dye concentration on the percentage removal of ARS onto DS and DSMNC is expressed in Figure 6B by plotting percentage removal vs. concentration. As portrayed in the figure, the percentage removal of ARS increases with increases in the initial dye concentration and reaches an optimum value (66.7% for DS and 97.89% for DSMNC) when the other parameters are kept constant. Furthermore, increasing the amount of dye beyond the optimum concentration led to saturation of the adsorption surfaces. This resulted in lowering the adsorption percentage. As the concentration of dye increases, more free dye particles are available to interact with the adsorbent, resulting in the formation of dye particle–adsorbent particle interactions, thereby enhancing the efficiency of dye removal. However, there comes a point where the dye removal efficiency may decrease or stabilize as the dye concentration reaches saturation (Ibrahim et al., 2019). There is an increase in the value of q_e_ in the pre-optimum value in a relatively steep manner, while after it reaches the optimum concentration, the curve flattens. The increase in the amount of dye creates a driving force for mass transfer, so the adsorbed dye amount per gram of adsorbent (q_e_, mg/g) increases.

The adsorbent dose and the percentage removal are directly related to the adsorption process (Elzahar and Bassyouni, 2023). Figure 6C illustrates the impact of the dose on the adsorption of ARS. With the rise in the quantity of the adsorbent, the percentage removal of dye also increases, reaching a maximum value of 76.6% for DS at 0.4 grams and 93.82% for DSMNC at 0.2 grams, after which it stabilizes. This trend can be credited to the expanded accessibility of adsorption sites with the higher adsorbent dosage, leading to enhanced dye–adsorbent interactions (Saha et al., 2011). There is a decrease in the q_e_ value with the increase in the adsorbent amount. This may be due to the fact that at the lower adsorbent amount, most of the adsorption sites are occupied by ARS molecules; hence, the amount of ARS per g of adsorbent increases, and increasing the amount of adsorbent may leave some of the adsorbent sites vacant at the same concentration.

Figure 6D illustrates the relationship between the percentage removal and shaking time, from 15 min to 150 min, for the adsorption process of alizarin red S onto both DS and DSMNC. The observed trend indicates a steady increase in percentage removal with increased shaking time, reaching its zenith at 60 min for DS and 45 min for DSMNC. Subsequently, a decline in removal efficiency is observed beyond these contact times. The variation of q_e_ with shaking time follows the same trend. This phenomenon is primarily attributable to the attainment of the adsorption–desorption equilibrium, wherein the surface coverage of the adsorbent reaches saturation at a given time interval. Further agitation beyond this equilibrium point leads to mechanical disruption of the already-adsorbed dye molecules, prompting desorption (Kuśmierek and Świątkowski, 2015). Moreover, comparing DS and DSMNC reveals that the latter exhibits a notably higher percentage removal value (increasing from 71.12% to 81.78%), indicating enhanced adsorption efficiency facilitated by the impregnation of magnetic nanoparticles, which provide the advantages of magnetic separation and increase in surface area (Rao and Ramanaiah, 2024). This augmentation underscores the role of magnetic NPs in boosting the adsorptive potential of the adsorbent.

Figure 6E portrays the impact of temperature variation on the percentage removal of alizarin red S by DS and DSMNC. The percentage removal increased from 55.12% at 20°C to 76.24% at 30°C, with a subsequent decrease in percentage removal with further temperature increments. Similar trends were observed for DSMNC, with a notably higher percentage removal of 86% at 30°C. This phenomenon can be attributed to the initial preferment of adsorption by temperature up to 30°C. Beyond this threshold, higher temperatures are hypothesized to favor dye diffusion in the solution phase rather than its adsorption onto the adsorbent (Khalaf et al., 2021). This inference aligns with the exothermic nature of the adsorption phenomenon and is supported by Le Chatelier’s principle, which posits that an increase in temperature tends to diminish the equilibrium constant (Kc) associated with adsorption, thereby disadvantaging the adsorption process (Abualnaja et al., 2021).

### 3.3 Adsorption kinetics study

The adsorption mechanism of alizarin red S onto DS and DSMNC was investigated through batch kinetic biosorption studies (Dehghani et al., 2017). Two 50 mL solutions of alizarin red S were subjected to shaking in the presence of 0.4 g of DS and 0.2 g of DSMNC, respectively, for varying time intervals ranging from 15 to 150 min while maintaining a steady temperature of 30°C throughout the adsorption process. The obtained adsorption data was scrutinized using pseudo-first-order, pseudo-second-order, and the intra-particle diffusion kinetics model equations given as follows:

Pseudo-first-order kinetics:

$$\ln\left(q_{e}-q_{t}\right)=\ln q_{e}-\;k_{1}t.$$ (3)

Pseudo-second-order kinetics:

$$\frac{t}{q_{t\;}}=\frac{1}{k_{2}q_{e}^{2}}+\frac{t}{q_{e}}.$$ (4)

Intra-particle diffusion model:

$$q_{t}=k_{dif}t^{1/2}+C.$$ (5)

Liquid diffusion model:

$$\ln\left(1-\frac{q_{t}}{q_{e}}\right)=-k_{fd}t.$$ (6)

In the given equations, q_e_ represents the equilibrium adsorption capacity (mg/g), q_t_ denotes the adsorption capacity at time t (mg/g), k_1_ is the pseudo-first-order equilibrium rate constant (min⁻^1^), k_2_ is the pseudo-second-order equilibrium rate constant (g/mg/min), k_dif_ is the rate constant of intra-particle diffusion, C is the intercept, and t is the adsorption time (min).

**FIGURE 7:**
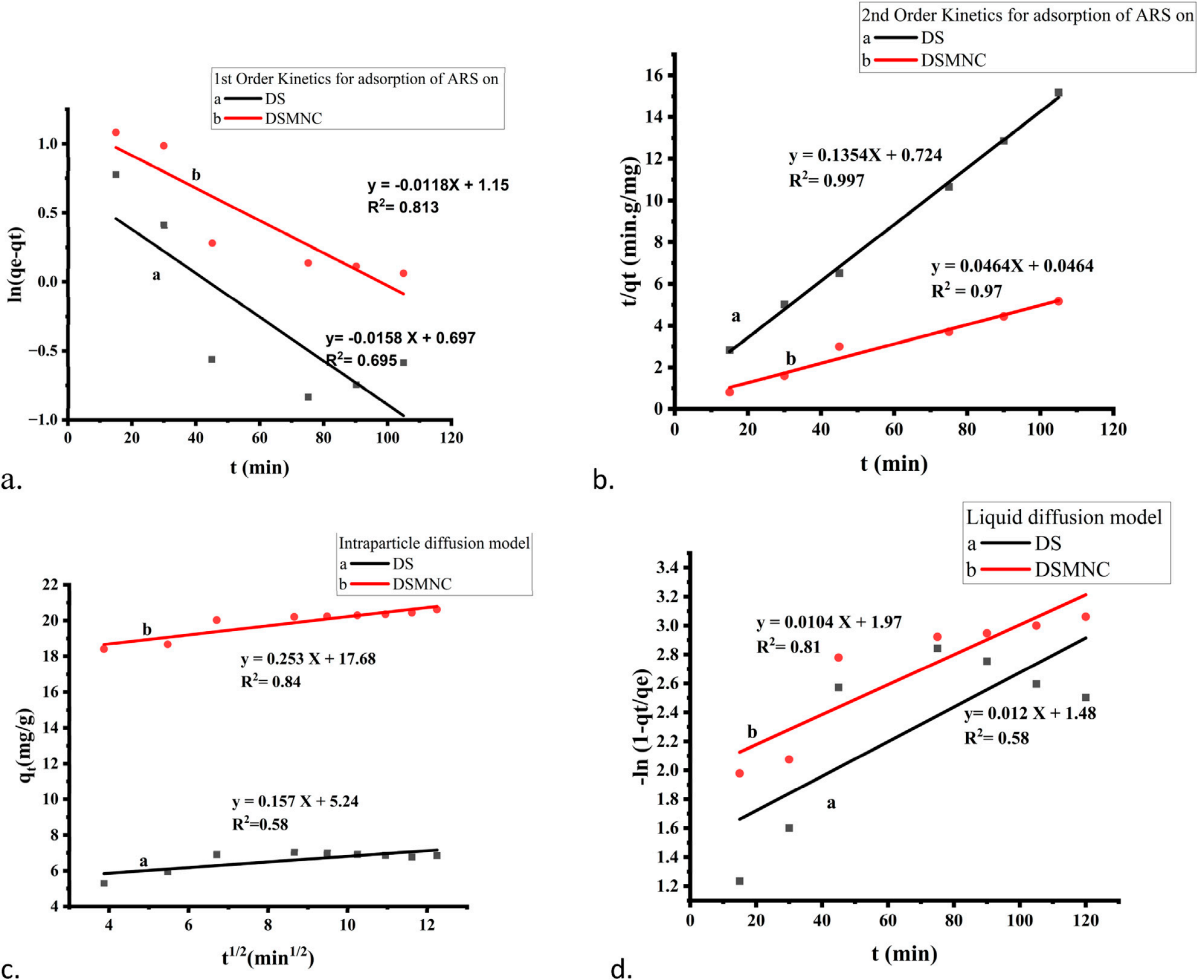
Kinetics models for the adsorption of ARS on DS and DSMNC: **(A)** pseudo-first-order, **(B)** pseudo-second-order, **(C)** interparticle diffusion model, and **(D)** liquid diffusion model.

The experimental values of q_t_ for both DS and DSMNC obtained during the shaking time experiment were subjected to fitting within the pseudo-first-order kinetic model (Equation 3). The resulting plot, depicted in Figure 7A, revealed that the *R* ^2^ values obtained from this analysis are 0.695 and 0.81, and values of specific rate constant (k) calculated from slope values are 1.6 × 10^−2^ min^−1^ and 1.19 × 10^−2^ min^−1^ for DS and DSMNC, respectively.

Figure 7B illustrates the adsorption behavior of alizarin red S onto the surface of DS and DSMNC over varying shaking intervals, with the data compiled in the pseudo-second-order kinetics (Equation 4). Calculation of the specific rate constants (k) from the slope of the curves reveals k values of 2.53 × 10^−2^ g. mg⁻^1^.min⁻^1^ for DS and 4.64 × 10^−2^ g·mg⁻^1^·min⁻^1^ for DSMNC. This signifies an enhancement in the adsorption rate upon the formation of magnetic nanocomposites from DS. The *R* ^2^ values for alizarin red S adsorption on DS and DSMNC are 0.997 and 0.97, respectively.

The value of q_e_ calculated from the intercept of PFO is 2.01 mg/g for DS and 3.16 mg/g for DSMNC, and from PSO, the values are 7.39 mg/g and 21.55 mg/g. The values obtained experimentally are 9.97 mg/g for DS and 21.35 mg/g for DSMNC, which are in close agreement with the values obtained from second-order kinetics, suggesting that the adsorption process for both DS and DSMNC obeys pseudo-second-order kinetics. This suggestion is also confirmed by a higher *R* ^2^ value for PSO, implying that dye molecules and various adsorption sites on a solid substrate randomly bump into each other during a rate-controlling mechanistic phase (Hubbe et al., 2019).

The study utilized both the intra-particle diffusion (Weber–Morris) model (Equation 5) and the liquid film diffusion model (Equation 6), as depicted in Figures 7C, D, to determine the mechanisms of diffusion for adsorption of ARS on DS and DSMNC. It was anticipated that liquid film diffusion, intra-particle diffusion, or a combination of both could serve as the limiting factors in the process (Ahmad et al., 2015). Weber and Morris observed that in many adsorption scenarios, the uptake of adsorbate is nearly linear to the square root of contact time rather than to the contact duration (Alkan et al., 2007). When intra-particle diffusion governs the adsorption process, a plot of q_t_ against t^1/2^ would be linear. Additionally, if this plot intersects the origin, it suggests that intra-particle diffusion is the only factor limiting the rate. On the other hand, if the straight-line graph of −ln (1 – q_t_/q_e_) vs. t has a zero intercept, it implies that the kinetics of adsorption are governed by diffusion via the liquid film neighboring the adsorbent (Hasani et al., 2022).

However, because neither intercept is equal to zero in this case, it is improbable that intra-particle diffusion alone dictates the rate-limiting step. Therefore, the kinetics were influenced by both liquid film and intra-particle diffusion simultaneously.

### 3.4 Isothermal study of adsorption

Various adsorption isotherm models are employed to comprehensively analyze experimental data and elucidate the adsorption mechanism of alizarin red S onto DS and DSMNC from aqueous solutions. These models, namely, Freundlich, Langmuir, Temkin, and Dubinin–Radushkevich (D–R), offer valuable insights into the interactions between alizarin red S molecules and the adsorbent surface.

The Freundlich isotherm:

$$logqe=\log k+\frac{1}{n}logCe.$$ (7)

The Langmuir isotherm:

$$\frac{1}{q_{e}}=\frac{1}{{q_{m}K}_{L}}+\frac{1}{q_{m}C_{e\;}}.$$ (8)

The Temkin isotherm:

$$q_{e}=\frac{RT}{b}\ln K_{T}+\frac{RT}{b}\ln C_{e}.$$ (9)

The D–R isotherm:

$$\ln q_{e}=\ln q_{m}-B\varepsilon^{2}$$ (10)

and

$$\varepsilon^{2}=RTln\left(1+1/C_{e}\right).$$ (11)

**FIGURE 8:**
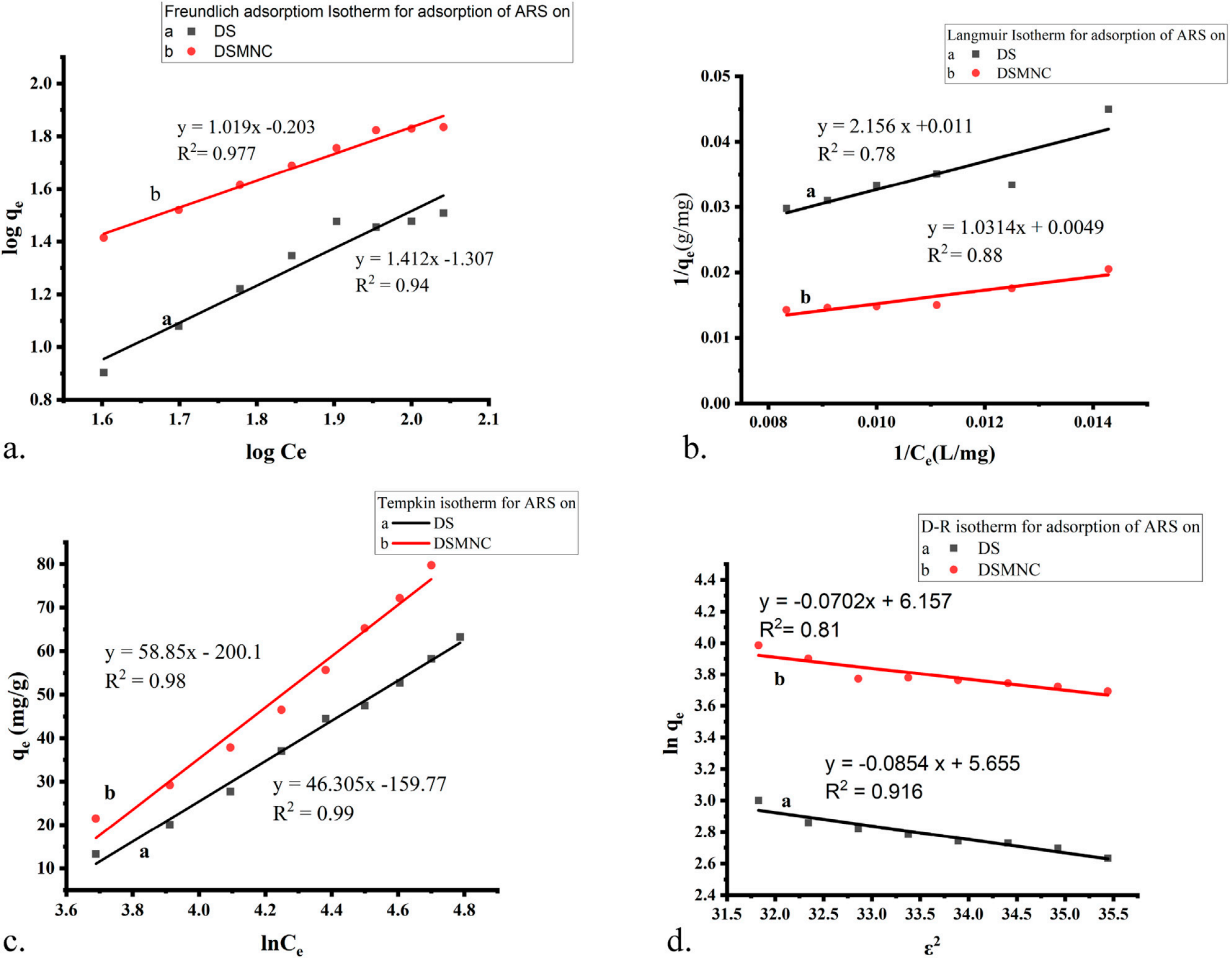
Adsorption isotherm models for the adsorption of ARS on DS and DSMNC: **(A)** Freundlich, **(B)** Langmuir, **(C)** Temkin, and **(D)** D–R adsorption isotherm.

By fitting experimental data to these various adsorption isotherm models shown in Figure 8 and analyzing the resulting parameters, such as equilibrium constants, adsorption capacities, and energy of adsorption, we can elucidate the adsorption mechanism and understand how alizarin red S molecules interact with the surfaces of DS and DSMNC. These insights are crucial for optimizing adsorption processes and designing efficient adsorbents for wastewater treatment or other applications.

Figure 8A depicts the Freundlich isotherm (Equation 7) plot for the adsorption of ARS on DS and DSMNC. The slope of the curve is 1.412 for DS and 1.019 for DSMNC, while the intercept is −1.307 for DS and −0.203 for DSMNC. The R-squared (*R* ^2^) values for the adsorption of the dye on both adsorbents are notably high: 0.977 for DS and 0.94 for DSMNC. These high *R* ^2^ values indicate a strong fit of the data to the Freundlich isotherm model. The calculated values of the Freundlich isotherm parameters, n, and K_F_, which characterize the adsorption behavior of ARS onto DS and DSMNC, are tabulated in Table 2 and provide insight into the affinity and capacity of the adsorbents for the dye.

**TABLE 1:** Summary of kinetics parameters obtained.

Adsorbent used	PFO kinetics			PSO kinetics			Interparticle diffusion model		Liquid film model	
Parameter	*R* ^2^	k_1_ × 10^−2^	q_e_	*R* ^2^	k_2_ × 10^−2^	q_e_	*R* ^2^	k	*R* ^2^	K
DS	0.695	1.6	2.01	0.997	2.53	7.39	0.58	0.157	0.58	0.0102
DSMNC	0.81	1.19	3.16	0.97	4.64	21.55	0.84	0.253	0.81	0.0104

**TABLE 2:** Values of Langmuir, Freundlich, Temkin, and D–R adsorption isotherm constants for ARS adsorption on DS and DSMNC powder.

Adsorbent used	Freundlich isotherm			Langmuir isotherm			Temkin isotherm			D–R isotherm		
	K_F_	n	*R* ^2^	q_m_ mg/g	K_L_ L/mg	*R* ^2^	K_T_ L/mol	b J/mol	*R* ^2^	q_max_ mol/g	E kJ/mol	*R* ^2^
DS	0.049	0.71	0.97	0.47	196	0.78	0.031	53.51	0.97	284	2.42	0.81
DSMNC	0.63	0.98	0.94	0.97	210	0.88	0.034	42.1	0.99	473	2.67	0.91

Figure 8B illustrates a plot depicting the Langmuir adsorption isotherm (Equation 8), showcasing straight lines with slopes of 2.156 and 1.013, along with intercepts of 0.011 and 0.005 for DS and DSMNC, respectively. The corresponding *R* ^2^ values stand at 0.78 and 0.88 for the adsorption of ARS onto DS and DSMNC, respectively. Utilizing the slope values, the maximum monolayer capacity (q_m_) is determined for DS and DSMNC. Notably, the monolayer capacity experiences a significant increase upon forming a nanocomposite of DS, attributable to the increased surface area resulting from particle size reduction, as confirmed by SEM analysis of the adsorbents. Langmuir constant (K_L_) is computed from the curve intercept for DS and DSMNC. Table 2 showcases the calculated q_m_ and K_L_ values acquired from the slope and intercept.

Figure 8C illustrates a plot depicting the Temkin isotherm (Equation 9) for the adsorption of ARS on both DS and DSMNC; the slopes of the lines are determined to be 46.30 and 58.85, respectively. These slopes indicate that physical adsorption is dominant in both cases. The values of the intercepts obtained from the linear plots are −159.77 and −200.1 for adsorption onto the DS and DSMNC surfaces, respectively. The Temkin isotherm constant (pertaining to the heat of adsorption) and equilibrium binding constants (K_T_) can be calculated from the value of slopes and intercepts. The calculated values of K_T_ for the adsorption of ARS on DS and DSMNC are summarized in Table 2.

These analyses demonstrate the applicability of the Temkin adsorption isotherm in describing the adsorption behavior of ARS onto both DS and DSMNC surfaces, with physical adsorption being the predominant mechanism (Dada et al., 2012).

The D–R isotherm (Equations 10, 11) is predominantly employed to determine the average adsorption-free energy (in kJ mol^−1^). It provides insights into adsorption behavior, suggesting a physical nature when the value falls within the limit of 1–8 kJ.mol^−1^ and a chemical nature when it resides between 8–16 kJ.mol^−1^ (Dehghani et al., 2017). The *R* ^2^ values derived from the plots are 0.91 and 0.81 for the adsorption of ARS onto DS and DSMNC, respectively, indicating a close correlation between the experimental data and the D–R isotherm model.

The average free energy of adsorption (E) is computed by analyzing the obtained values of B from the slope, −0.0853 and −0.0702 for DS and DSMNC, respectively. The result indicates that the adsorption process for both adsorbents is physical in nature. The intercepts, having values of 5.65 and 6.16, are utilized to calculate q_m_, the maximum adsorption capacity, for the adsorption of ARS on the surfaces of DS and DSMNC, respectively.

### 3.5 Adsorption thermodynamics study

Thermodynamic studies of dye biosorption are often conducted (using Equations 12–15) to ascertain the viability of the sorption process. Three key exploratory parameters are the standard entropy change (ΔS), the standard enthalpy change (ΔH), and the Gibbs free energy change (ΔG) (Bilal et al., 2022).

$${∆G=-RTlnK}_{c},$$ (12)

$$K_{c}=\frac{C_{ad}}{C_{after}},$$ (13)

∆G=∆H−T∆S, (14)

$$\ln K_{c}=\frac{∆S}{R}-\frac{∆H}{RT}.$$ (15)

**FIGURE 9:**
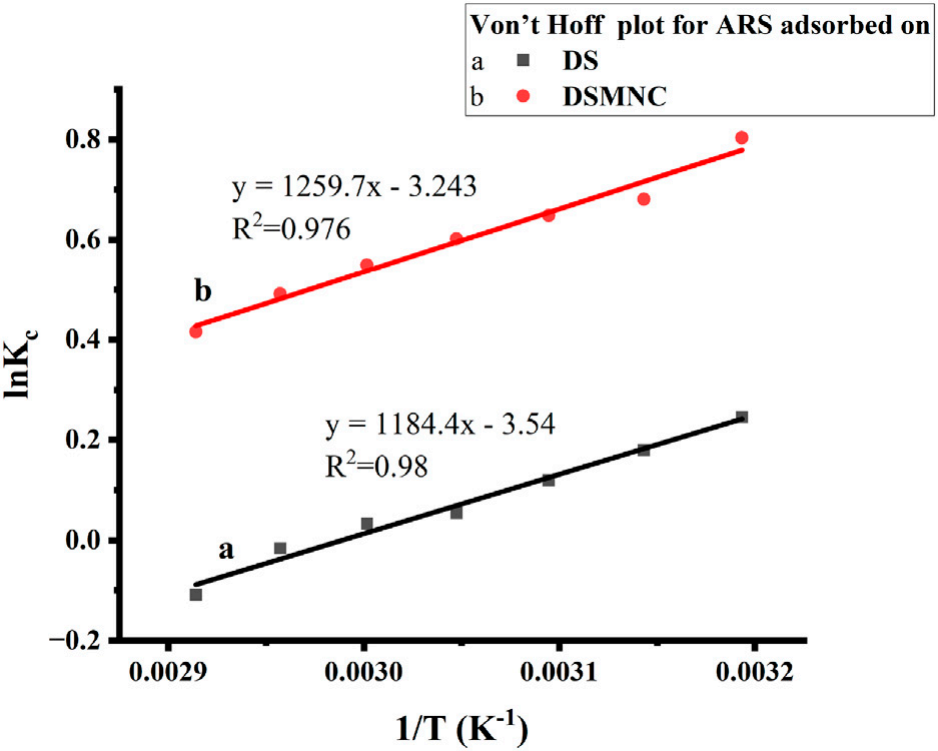
Thermodynamics study of adsorption of ARS on DS and DSMNC.

Figure 9 depicts a thermodynamic study of the adsorption of alizarin red S on DS and DSMNC at various temperatures.

By plotting ln K_c_ against 1/T, a linear relationship (Equation 15) is observed, yielding slopes of 1,184.4 and 1,259.7, with intercepts of −3.54 and −3.25, respectively. The high values of *R* ^2^ (0.9834 and 0.9767) indicate an excellent correlation between the adsorption data (Elmorsi, 2011). From the slopes, ΔH is calculated to be −9.85 kJ/mol and −10.47 kJ/mol for the adsorption of alizarin red S on DS and DSMNC, respectively. The intercepts give the ΔS as −29.43 J/mol and −26.97 J/mol for DS and DSMNC, respectively. Equation 14 yields the values of ΔG energy as −1072 J/mol and −2427 J/mol for the adsorption of alizarin red S on DS and DSMNC, respectively. These calculations indicate that the process is physical adsorption, enthalpy-driven, and spontaneous (Ebelegi et al., 2020).

## 4 Conclusion

In the current investigation, we addressed the pressing environmental issue of non-biodegradable organic dyes by exploring the adsorption characteristics of ARS using DS and a DSMN composite. Batch adsorption experiments demonstrated a maximum removal efficiency of 76.63% for DS and 97.89% for DSMNC at 30°C, indicating favorable adsorption onto the heterogeneous surface of the composite. This adsorption process was facilitated by a combination of weak interactive forces. These outcomes suggest that both DS and DSMNC are potent for the removal of the mutagenic dye, ARS, with DSMNC exhibiting higher removal efficiency.

## Data Availability

The datasets presented in this study can be found in online repositories. The names of the repository/repositories and accession number(s) can be found in the article/Supplementary Material.
