# Supplementary material for: Kinetics and thermodynamics investigations of efficient and eco-friendly removal of alizarin red S from water via acid-activated Dalbergia sissoo leaf powder and its magnetic iron oxide nanocomposite
Source: Front Chem. 2024 Sep 25;12:1457265. doi: 10.3389/fchem.2024.1457265 (PMC11462623; doi:10.3389/fchem.2024.1457265)
Supplement: Supplementary file 1 [file Presentation1.pdf]

Cost-effective and eco-friendly removal of Alizarin red S from aqueous medium via acid-activated powdered Dalbergia Sissoo leaves and its magnetic iron-oxide nanocomposite

Saleem Nawaz [a], Syed Muhammad Salman[a],Asad Ali*[b], Basit Ali[a], Syed Nusrat Shah[a],

Latif Ur Rahman[c],

[a.] Department of Chemistry, Islamia College Peshawar, Pakistan 25120,

[b.]Energy Engineering, Division of energy Science, Lulea university of Technology, 97187, Lulea, Sweden.

[c] Institute of Chemical Sciences, University of Peshawar, Pakistan 25120,

Corresponding Author: asad.ali@asssociated.ltu.se (A. Ali)

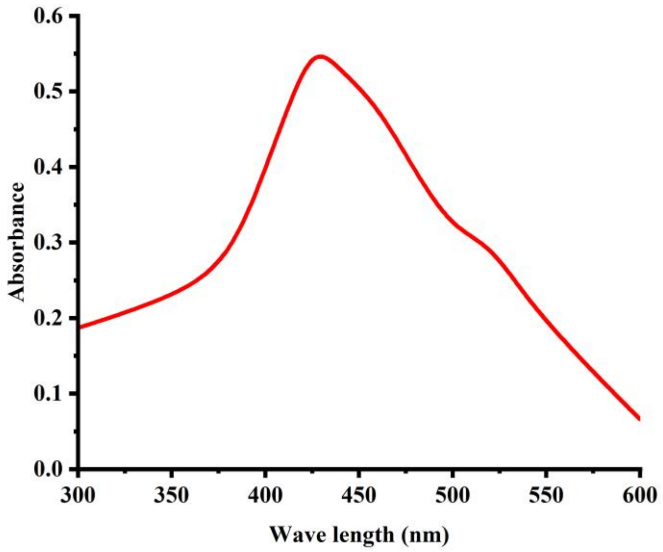

Fig. S1 Plot of absorbance of ARS at different wavelength

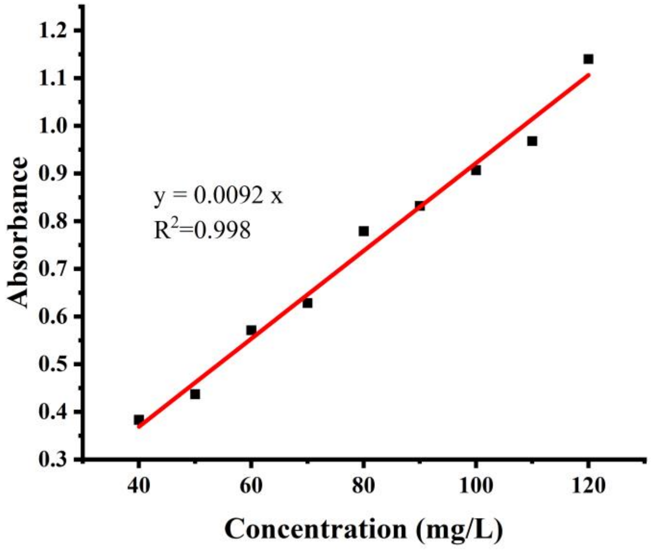

Fig. S2 Plot of absorbance vs concentration of ARS

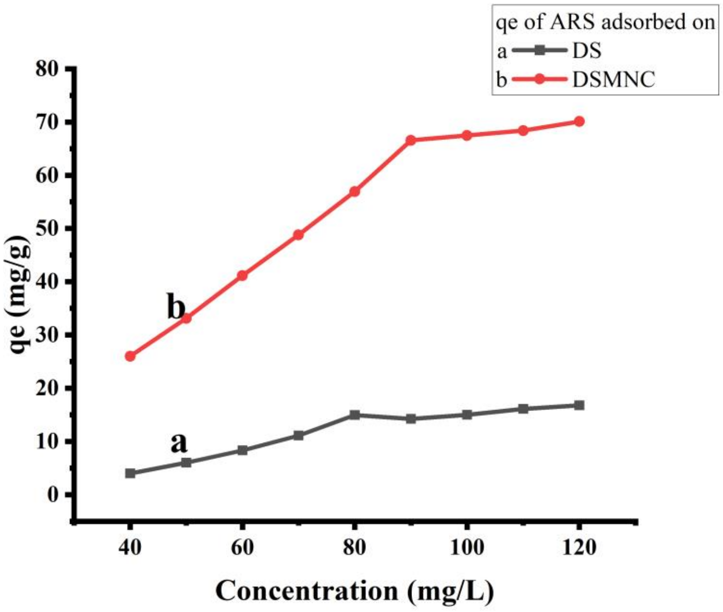

Fig. S3 Effect concentration on Adsorption capicity(qe) of ARS on DS and DSMNC

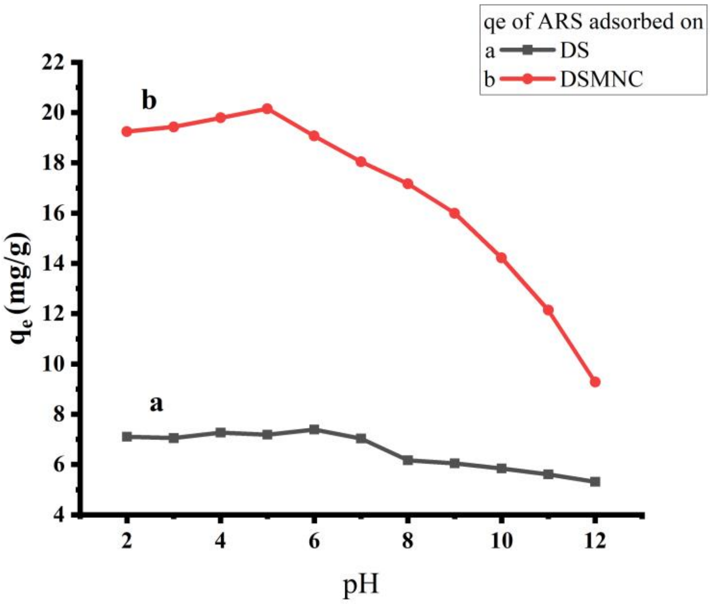

Fig.S4 Effect of pH on qe of adsorption of ARS on DS and DSMNC

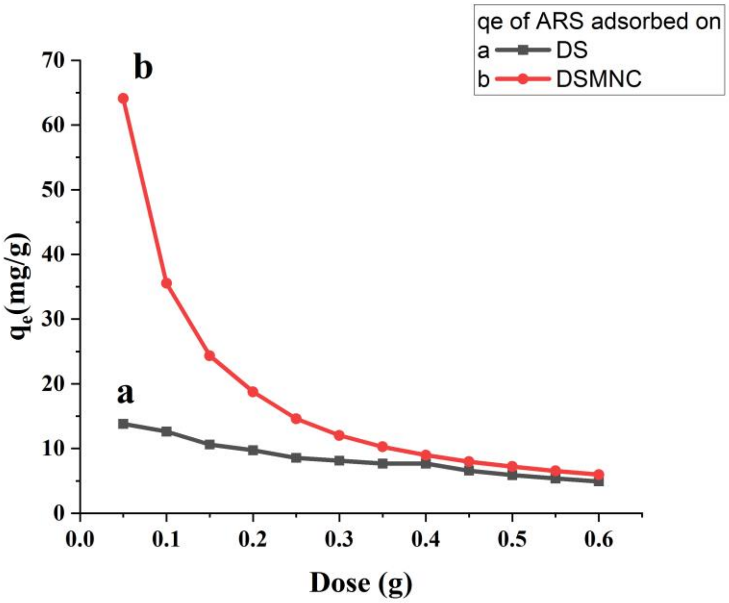

Fig.S5 Effect of adsorbent dose on qe of ARS adsorbed on DS and DSMNC

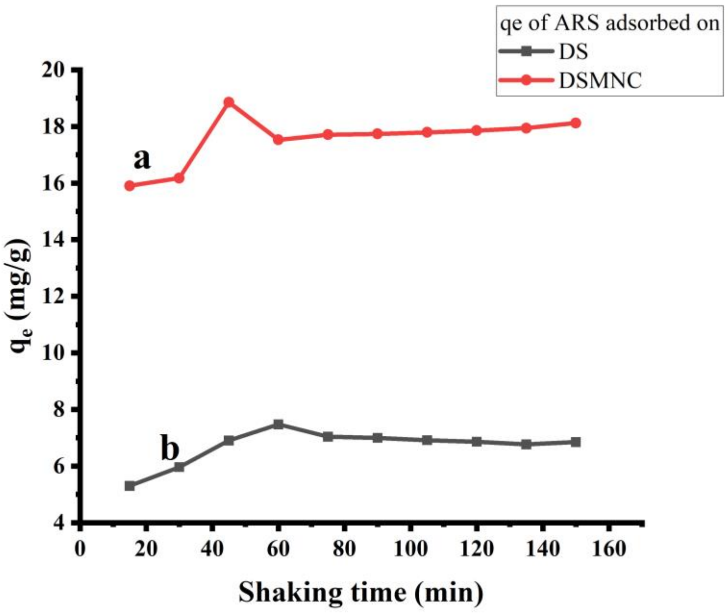

Fig.S6 Effect of shaking time on qe of ARS adsorbed on DS and DSMNC

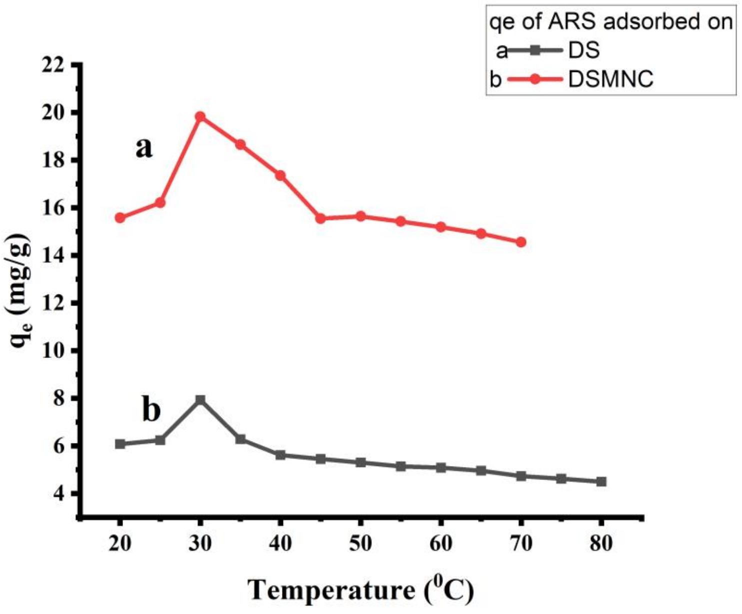

Fig. S7 Effect of Temperature on q_e of ARS adsorbed on DS and DSMNC
